# How air pollution influences the difference between overweight and obesity: a comprehensive analysis of direct and indirect correlations

**DOI:** 10.3389/fpubh.2024.1403197

**Published:** 2024-11-01

**Authors:** Muchun Yu, Jinchen Xie, Yanyan Liu

**Affiliations:** ^1^Department of Philosophy, School of Humanities and Social Sciences, Xi’an Jiaotong University, Xi’an, China; ^2^Global Health Institute, School of Public Health, Xi’an Jiaotong University, Xi’an, China

**Keywords:** obesity, air pollutants, determinants, regression discontinuity, structural equation modeling

## Abstract

**Background:**

Obesity, characterized by excessive or abnormal fat accumulation, is a major public health concern. Air pollution is a significant potential obesogenic factor, but the clear direct and indirect correlations between air pollution and obesity remain unclear. This study aims to provide a comprehensive understanding of the relationship between air pollution and obesity by identifying both direct and indirect causal correlations.

**Methods:**

We used nationally representative data from the China Family Panel Survey. Air pollution concentrations were quantified as the mass (μg) of air pollutants per cubic meter (m^3^) based on nationally representative statistical data. To minimize statistical bias inherent in traditional methods, the direct relationship between air pollution and obesity was estimated using a regression discontinuity model, while the potential underlying mechanisms were explored through structural equation modeling.

**Results:**

Air pollution was generally positively associated with overweight/obesity ( $OR_{OW}^{AQI}$ = 1.109, [95%CI = 1.027:1.305], $OR_{OB}^{AQI}$ = 1.032, [95%CI = 1.006:1.217], $OR_{\mathrm{SO}}^{AQI}$ = 1.069, [95%CI = 1.014:1.208], PM_2.5_ and PM_10_ positively affected overweight/obesity ( $OR_{OW}^{\mathrm{PM}2.5}$ = 1.173, [95%CI = 1.094:1.252], $OR_{OB}^{\mathrm{PM}2.5}$ = 1.022, [95%CI = 1.016:1.028], $OR_{\mathrm{SO}}^{\mathrm{PM}2.5}$ = 1.035 [95%CI = 1.015:1.055], $OR_{OW}^{\mathrm{PM}10}$ = 1.053, [95%CI = 1.030:1.076], $OR_{OB}^{\mathrm{PM}10}$ = 1.008 [95%CI = 1.006:1.010], $OR_{\mathrm{SO}}^{\mathrm{PM}10}$ = 1.013 [95%CI = 1.007:1.019]), and SO_2_ and CO posed negative impacts on overweight/obesity ( $OR_{OW}^{\mathrm{SO}2}$ = 0.972, [95%CI = 0.965:0.979], $OR_{OB}^{\mathrm{SO}2}$ = 0.997, [95%CI = 0.996:0.998], $OR_{\mathrm{SO}}^{\mathrm{SO}2}$ = 0.994, [95%CI = 0.991:0.997], $OR_{OW}^{CO}$ = 0.986, [95%CI = 0.980:0.992], $OR_{OB}^{CO}$ = 0.998, [95%CI = 0.997:0.999], $OR_{\mathrm{SO}}^{CO}$ = 0.999, [95%CI = 0.998:0.999]). The impact of air pollution on overweight/obesity was more significant among men, older individuals, and rural populations compared to women, younger individuals, and urban populations. Furthermore, the relationship between air pollution and overweight/obesity was mediated by social behavior determinants, including physical activity (*β* = 0.18, [95%CI = 0.04:0.29]), sedentary behavior (*β* = 0.12, [95%CI = 0.04:0.16]), sleep (β = 0.06, [95%CI = 0.02:0.13], smoking (β = 0.07, [95%CI = 0.02:0.15]), alcohol consumption (β = 0.08, [95%CI = 0.04:0.11]), and mental health (β = 0.06, [95%CI = 0.01:0.09]).

**Conclusion:**

Air pollution was generally associated with an increased risk of overweight and obesity, with PM_2.5_ and PM_10_ having a positive influence, while SO_2_ and CO had a negative impact. The effect of air pollution was more pronounced among men, older individuals, and rural populations compared to women, younger individuals, and urban populations. Additionally, social behavior factors, such as physical activity, sedentary behavior, sleep, smoking, alcohol consumption, and mental health, predominantly mediated the relationship between air pollution and obesity.

## 1 Introduction

Overweight and obesity (OW/OB), characterized by excessive and abnormal accumulation of fat, have become major threats to public health worldwide (1–4). The prevalence of OB has reached pandemic proportions, affecting over 650 million adults and 340 million adolescents globally (5–7). The strong correlation between OW/OB and chronic diseases like type 2 diabetes, hypertension, liver cancer, colon cancer and myocardial infarction has been widely proven (4, 8–10). Moreover, OB is also associated with psychological factors such as self-esteem, depression, and weight-based stigma, negatively impacting life expectancy and general wellbeing (7, 11). Additionally, the global medical costs associated with OW/OB treatments are projected to rise to 300 billion dollars by 2030, accounting for a staggering 21.5% of total healthcare expenditures (7, 11, 12).

Given the significant impact of OW/OB on public health, identifying key determinants has become an urgent priority. While traditional factors such as diet, exercise, and sleep have been extensively studied (13–17), the relationship between air pollution and OW/OB remains underexplored.

Air pollution, which consists of harmful compounds in the atmosphere emitted by both natural and human sources, including particulate matter, carbon oxides, sulfur oxides, and nitrogen oxides, has been identified as a potentially important determinant of several diseases (5, 10, 13). According to the WHO, air pollution and OW/OB rank as the second and third most hazardous determinants of mortality (5). Given these alarming statistics, the joint prevention and control of air pollution and OW/OB has become a critical priority, particularly in line with the UN General Assembly High-Level Meeting’s political declaration on disease prevention and control efforts (5, 10, 13).

From an academic perspective, the causal relationship between air pollution and OW/OB has not been fully established (6, 18). Several empirical studies have demonstrated that improved air quality can contribute to weight loss by reducing the respiratory burden and mitigating endocrine disorders (5, 19, 20). However, some studies have found that air pollution has an insignificant influence on OW/OB. For instance, Furlong and Klimentidis reported a statistically insignificant relationship between nitrogen oxides and obesity (21), and Yu concluded that PM exposures were unrelated to obesity (22). Similar findings were reported by other interdisciplinary scholars (19).

This conflicting evidence is further highlighted in a meta-analysis that examined the correlation between air pollutants and OW/OB, which found that 44% of the studies showed a positive correlation, 44% showed no correlation, and 12% showed a negative correlation (23). This controversy likely stems from the fact that prior research focused solely on correlations rather than causality, resulting in statistical biases such as sample self-selection, omitted variables, and reverse causation, which have affected the accuracy of the estimates (2, 4, 6).

Moreover, another significant academic gap in prior research is the unclear potential mechanisms linking air pollution and OW/OB, especially from a social behavior perspective (2, 4, 6, 21, 24). Social behavior, defined as the “aggregate of behaviors produced by individuals based on their specific social class,” is recognized by sociologists as a key determinant of health disparities (25). Incorporating social behavior into obesity research aligns with the bio-socioecological framework for obesity analysis, as outlined in “The Lancet” (26). However, prior studies have largely overlooked the potential mechanisms through which air pollution impacts obesity, as mediated by social behavior factors. In the present study, we address this gap by including six mediators: physical activity, sleep duration, smoking, alcohol consumption, mental health, and sedentary behavior. These factors are not only influenced by air pollution but are also important determinants of obesity (11, 27, 28).

Taken together, two major academic gaps have been identified in previous analyses of the relationship between air pollution and OW/OB: the lack of accurate causal analyses and the limited exploration of potential mechanisms related to social behavior. To address these gaps, we isolate the net direct association between air pollution and OW/OB using a regression discontinuity (RD) model based on an arbitrary Chinese heating policy. Additionally, we explore the indirect relationship between air pollution and OW/OB through the mediation of social behavior factors. The following three questions are discussed: (1) What is the causal relationship between air pollution and obesity? (2) What is the indirect correlation between air pollution and obesity, mediated by social behavior factors? (3) How do these relationships vary across different subgroups?

We chose only Chinese adults aged 18–60 because they are more exposed to air pollution exposure due to outdoor work and represent the group most severely affected by obesity (11, 29).

## 2 Methodology

### 2.1 Study design and subjects

The present study utilized nationally representative large-scale data from the China Family Panel Survey (CFPS), a comprehensive database conducted by Peking University. The CFPS provides robust data on respondents’ geographic location, body features, and demographic characteristics, offering substantial evidence to analyze the relationship between air pollution and OW/OB. The 2016, 2018, and 2020 rounds of the CFPS were selected because they covered all 31 Chinese provinces.

Initially, the dataset included 86,335 respondents. The following pre-processing steps were performed: First, respondents with missing geographic location, height, weight, or demographic characteristics were removed, yielding 37,825 respondents. Second, we retained only individuals aged 18–60 years, resulting in 32,711 respondents. Third, we excluded respondents with incorrectly entered data (e.g., height ≤ 50 cm or ≥ 300 cm and weight ≤ 20 kg or ≥ 300 kg), leaving 32,158 respondents for analysis. This study was approved by the Institutional Ethics Committee of Xi’an Jiaotong University.

### 2.2 Key variables

#### 2.2.1 Air pollutants concentrations

Air pollutant concentrations (APCs) were measured as the mass (μg) of air pollutants per cubic meter (m^3^), including PM_2.5_, PM_10_, SO_2_, CO, NO_2_, and O_3_. To provide a comprehensive view of the relationship between air pollution and obesity, we also generated the Air Quality Index (AQI). We determined the APC data for each respondent through the following steps:

(1) Identifying the respondent’s residence. Geographic information for respondents’ residences was obtained using the China Geographic Information Public Service Platform.^1^

(2) Constructing the APC database. We built an APC database for each respondent by integrating data from the Chinese Environmental Statistical Yearbook (CESY) and Chinese City Air Quality Monthly Reports (CCAQMR). APC information for each respondent was calculated on a city-by-city basis. Detailed steps for generating APCs can be found in the Supplementary Data 1.

(3) Balancing potential bias. APC data for PM_2.5_, PM_10_, SO_2_, NO_2_, and CO were weighted by their daily average values, while for O_3_, the 8-h average daily maximum concentration was used. All six APCs were measured in strict accordance with the International Reference Method Criteria (IRMC) (30). To account for respondents who may have moved during the interview year, APC data were time-weighted based on their different residences. To improve estimation accuracy, we employed parcel-level data in an inverse distance-squared weighting algorithm, which spatially interpolated air quality data from up to four monitoring stations within a 50 km radius of each participant’s residence (18).

(4) Generating the AQI index. The AQI was calculated to assess the air pollution status in each region. The AQI determines whether the six APCs meet or exceed limit values, with higher scores indicating more severe air pollution (30, 31). Additional details on the AQI calculation process are shown in Supplementary Data 1 and Supplementary Table S2.

Notably, we selected six APCs and the AQI to report simultaneously to capture a comprehensive view of the relationship between air pollution and OW/OB. We obtained daily information on the concentrations of the six pollutants for the CFPS-validated cities for the period 2016–2020. Pollutant concentrations averaged over the CFPS survey months (July of each year) were then calculated by averaging the mean values. The AQI calculations were based on the following time frames: (1) PM10: 24-h, (2) PM2.5: 24-h, (3) NO2: 1-h, (4) O3: 8-h, (5) SO2: 1-h, and (6) CO: 8-h (30, 31). More details are provided in the section Supplementary Data 1.

#### 2.2.2 Obesity outcomes

We focused on three OW/OB categories: OW, OB, and severe obesity (SO). Body Mass Index (BMI) was used to assess these states, calculated as weight (kg) divided by height squared (m^2^). According to the recommendations of the WHO, we defined BMI ≥ 24 as OW, BMI ≥ 28 as OB, and BMI ≥ 30 as SO (13, 28, 32). In the full sample, there were 12,236 individuals with OW, 2,873 with OB, and 444 with SO.

#### 2.2.3 Covariates

The covariates in the present study included age, gender (0 = women, 1 = men), rural residence (0 = urban, 1 = rural), wage, education, employment status (0 = unemployed, 1 = employed), physical activity (PA), sleep duration, smoking status (0 = non-smoker, 1 = smoker), alcohol consumption (0 = non-drinker, 1 = drinker), mental health (MH), sedentary behavior (SB), temperature, and wind. Additionally, PA, sleep, smoking, alcohol consumption, MH, and SB were considered mediators to capture the indirect correlation between air pollution and OW/OB from a social behavior perspective (25). All selected variables and their operationalizations are detailed in Supplementary Table S3.

### 2.3 Statistical analysis

First, the chi-square test (for categorical variables) and the t-test (for continuous variables) were used to identify disparities in respondents’ obesity outcomes, APC data, and other covariates across different samples.

Second, the RD model was used to explore the causal relationship between air pollution and OW/OB/SO. The RD model in the present study was based on an arbitrary Chinese heating policy, the Qin Huai boundary (QHB) policy. The QHB policy provided free heating for northern Chinese residents but not for those in the south, resulting in significant fossil fuel combustion in the north, which resulted in substantial air pollution and associated health risks.

Importantly, residents on either side of the QHB, within a relatively close distance, shared similar socioeconomic characteristics. This created quasi-randomized groups: the control group, located in the south with lower air pollution, and the treatment group, located in the north with higher pollution levels. Both groups were otherwise consistent in key socioeconomic determinants of OW/OB (33). More details about the QHB policy can be found in Supplementary Data 2. The RD model used in the analysis is represented as shown in Equations 1 and 2:


(1) $$\begin{array}{l} APCs_{i,t}=\alpha_{0}+\alpha_{1}D_{i}+\alpha_{2}f\left(L_{i}\right)+\alpha_{3}D_{i}*f\left(L_{i}\right)\\ +\mathrm{\delta COV}s_{i,t}+\nu_{i,t} \end{array}$$

(2) $$\begin{array}{l} O_{i,t}=\beta_{0}+\beta_{1}APCs_{i}+\beta_{2}f\left(L_{i}\right)+\beta_{3}D_{i}*f\left(L_{i}\right)\\ +\mathrm{\varphi COV}s_{i,t}+\mu_{i,t}\text{,} \end{array}$$

where APCs_i,t_ was APCs for individual i in region t. O_i,t_ denoted the situation of OB/OB for individual i in region t, which took a value of 1 to indicate the OW/OB, while a value of 0 indicated none. D_i_ was a dummy variable indicating the relative position of the individual to QHB (=1 for located QHB north, = 0 for south). f(L_i_) denoted the fitting polynomial order. D_i_*f(L_i_) was an interaction term that balanced the heterogeneous influence in QHB North–South by the polynomial, $\nu_{i,t}$ , $\mu_{i,t}$ denoted residuals. Each residence and its QHB distance (100 KM) information are shown in Supplementary Table S1.

Third, we analyzed the potential mechanisms using structural equation modeling (SEM), where the dependent variables were OW/OB/SO, the independent variable was AQI, and the mediators included PA, sleep, smoke, alcohol, MH, and SB. The initial SEM model is shown in Supplementary Figure S1. A modification index was utilized to optimize the models, and bootstrap sampling was repeated 5,000 times to ensure robustness. Potential bias was addressed through RD analysis.

The RD model was used to assess the causal association between air pollution and OW/OB, while the SEM model was used to reveal the potential mechanism between air pollution and OW/OB. All analyses were conducted using R version 4.2 and Mplus version 8.2.

## 3 Results

### 3.1 Data description

Table 1 systematically presents the distribution of variables across the full sample (first column), the northern sample (second column), the southern sample (third column), the subgroup differences (fourth column), the subgroup differences after adjusting for the QHB distance cubed (fifth column), and the significance test of these differences (sixth column). The following descriptive conclusions can be drawn: (1) the prevalence of OW, OB, and SO, as well as the AQI and different pollutant concentrations, differed significantly between South and North China, regardless of whether they were adjusted by the QHB distance; (2) the covariates differed significantly across subgroups before balancing the QHB distance but became insignificant after the QHB distance adjustment.

**Table 1:** Overweight, obesity, and severe obesity outcomes, air pollutants information and other characteristics of the Chinese adults in the China Family Panel Survey.^a,b,c^

Variables	Full sample (32,158)	North sample (13,632)	South sample (18,526)	Subgroup differences^e^	Adjust subgroup differences^d,e^	*P*-value^b^
Panel 1: Physical characteristics						
OW^c^	38.05%	42.08%	32.57%	9.5%***	7.32%***	<0.01/<0.01
OB^c^	8.93%	10.63%	6.63%	4%***	3.55%***	<0.01/<0.01
SO^c^	1.38%	1.72%	0.92%	0.8%***	0.68%***	<0.01/<0.01
Panel 2: Air pollution information						
AQI	4.20 (0.01)	4.82 (0.01)	3.34 (0.01)	1.48***	1.22***	<0.01/<0.01
PM_2.5_	37.39 (0.08)	44.46 (0.09)	27.26 (0.07)	17.15***	15.17***	<0.01/<0.01
PM_10_	49.49 (0.13)	56.63 (0.18)	39.24 (0.14)	17.05***	14.48***	<0.01/<0.01
SO_2_	38.03 (0.23)	51.89 (0.34)	18.13 (0.13)	33.77***	28.26***	<0.01/<0.01
CO	1.83 (0.01)	2.22 (0.01)	1.27 (0.01)	0.95***	0.74***	<0.01/<0.01
NO_2_	42.32 (0.11)	47.73 (0.15)	34.56 (0.15)	13.17***	11.25***	<0.01/<0.01
O_3_	100.79 (0.31)	94.75 (0.44)	109.47 (0.42)	14.72***	12.36***	<0.01/<0.01
Panel 3: Demographic characteristics						
Age	38.28 (0.16)	38.63 (0.21)	38.05 (0.23)	0.83***	0.12	<0.01/0.33
Men	51.08%	51.44%	50.43%	1.01%	0.44%	0.07/0.68
Rural	25.52%	25.12%	26.07%	−0.95%	−0.27%	<0.01/0.75
Wage	10.68 (6.26)	10.24 (0.02)	10.48 (0.02)	−0.19***	−0.08	<0.01/0.78
Edu	11.84 (0.05)	11.91 (0.07)	11.77 (0.08)	0.11**	0.04	0.04/0.84
Employed	83.83%	82.87%	85.14%	−2.27%***	−1.09%	<0.01/0.69
Smoke	28.89%	29.73%	27.76%	1.97%***	0.52%	<0.01/0.77
Alcohol	11.61%	13.27%	12.85%	1.31%	0.79%	<0.01/0.82
PA	95.97 (2.01)	100.11 (2.69)	89.93 (2.99)	1.48	0.65	0.57/0.89
Sleep duration	7.47 (0.01)	7.12 (0.04)	7.95 (0.07)	0.24***	0.01	<0.01/0.33
MH	2.22 (0.01)	2.21 (0.01)	2.24 (0.01)	0.029	0.013	0.13/0.49
SB	3.66 (0.01)	3.67 (0.01)	3.61 (0.02)	0.055***	0.011	<0.01/0.19
Tem	1.61 (0.12)	−3.81 (0.14)	7.94 (0.11)	12.16***	4.28	<0.01/0.63
Wind	2.76 (0.01)	2.61 (0.02)	2.93 (0.02)	−0.29***	−0.08	<0.01/0.72

^a^The χ2 test and *t*-test were used for categorical variables (overweight (OW), obesity (OB), severe obesity (SO), female (0 = female, 1 = male), rural (0 = urban, 1 = rural), smoking (0 = non-smoking, 1 = smoking), alcohol (0 = non-alcohol, 1 = alcohol) and continuous variables (air quality index (AQI), PM_2.5_, PM_10_, SO_2_, CO, NO_2_, O_3_, age, wage, education, sleep duration, mental health (MH), sedentary behavior (SB), temperature, wind), respectively. ^b^*p < 0.05, **p < 0.01, ***p < 0.001. ^c^Individuals were defined as OW, OB, and SO if their BMI ≥ 24, BMI ≥28, and BMI ≥30, respectively. ^d^Subgroup difference was the northern sample mean minus the southern sample mean for a given variable, e.g., the subgroup difference for OW is the OW _north_ (42.08%) - OW _south_ (32.57%) = 9.5%. The significance was the different distribution of a given variable in the South and the North. ^e^Adjust difference was referred to as the adjustment of subgroup difference by the cubic of QHB distance. The significance was the different distribution of a given variable in the South and the North.

### 3.2 The direct relation estimation: RD analysis

#### 3.2.1 Pre-testing: RDplot

**Figure 1:**
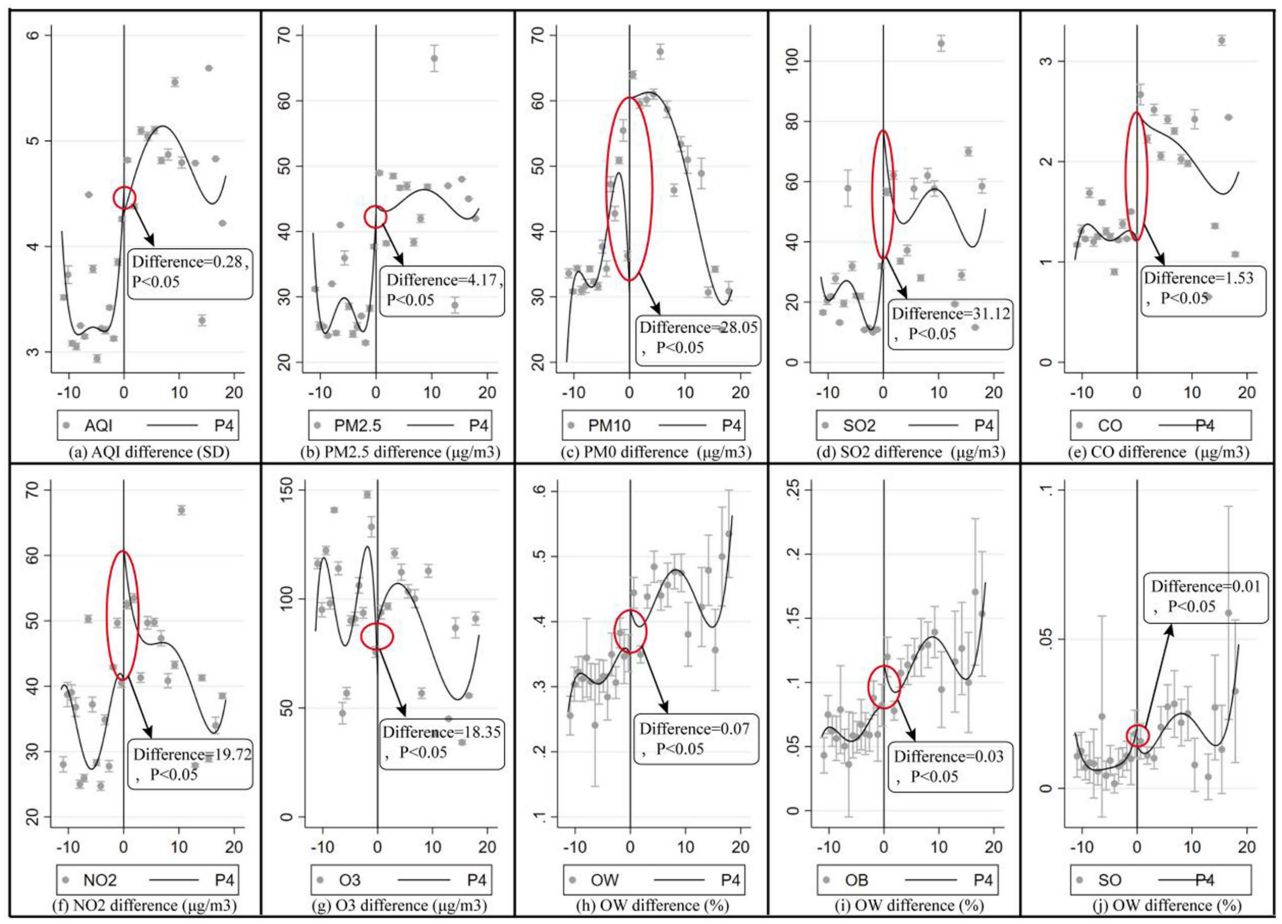
RD plot: discontinuous changes of selection variables on both sides of the QHB. The result was shown through the ‘RD plot’ command in the STATA 16.0; the significance of differences was tested through the *t*-test, P4 denoted polynomial = 4.

The “RDpolt” procedure was used to examine whether there were discontinuous changes in OW, OB, SB, and APCs around the QHB and to verify the suitability of the selected sample for RD analysis. The results are shown in Figure 1. According to the “Akaike Information Criterion (AIC) principle,” we chose to report the results for polynomial order = 4. Additional information is shown in Supplementary Figures S2–S4.

Figures 1a–g show the discontinuous variations in AQI, PM_2.5_, PM_10_, SO_2_, CO, NO_2_, and O_3,_ with respective differences of 0.28, 4.823 μg/m^3^, 28.744 μg/m^3^, 41.024 μg/m^3^, 19.752 μg/m^3^, 1.366 μg/m^3^, and 26.355 μg/m^3^ at the QHB. Figures 1h–j reveal statistically significant discontinuous changes of 7.32, 3.14, and 0.06% in the prevalence of OW, OB, and SB, respectively, at the QHB. Taken together, these results confirm that the data meet the conditions for RD analysis, as statistically significant discontinuous changes in both the independent and dependent variables were observed at the QHB breakpoint.

#### 3.2.2 The direct relation estimation: RD analysis

Table 2 presents the direct causal relationship between APCs and OW/OB/SO based on RD analysis. According to the “AIC principle,” we report the RD model results with a bandwidth of 2 and a polynomial order of 4. Additional details are available in Supplementary Table S4. Overall, air pollution was positively correlated with the prevalence of OW/OB/SO. Specifically, a 1-unit increase in AQI was associated with statistically significant increases of 10.9% (OR = 1.109, 95%CI = 1.027–1.305) in OW, 3.2% (OR = 1.032, 95%CI = 1.006–1.217) in OB, and 6.9% (OR = 1.069, 95%CI = 1.014–1.208) in SO morbidity.

**Table 2:** Causal direct correlation (odds ratio and 95% CI) between air pollutants and the morbidity of overweight, obesity, and severe obesity^a^: RD estimates.^b^

Variables	AQI	PM_2.5_	PM_10_	SO_2_	CO	NO_2_	O_3_
Panel 1: Air pollutants on overweight (*d* = 2, polynomial = 3)^c^							
OR(OW)	1.109***	1.173***	1.053***	0.972***	0.986***	1.052	1.026
95%CI	1.027–1.315	1.094–1.252	1.030–1.076	0.965–0.979	0.980–0.992	0.982–1.092	0.989–1.043
Panel 2: Air pollutants on obesity (*d* = 2, polynomial = 3)^c^							
OR(OB)	1.032***	1.022***	1.008***	0.997***	0.998***	1.000	1.001
95%CI	1.006–1.217	1.016–1.028	1.006–1.010	0.996–0.998	0.997–0.999	0.998–1.002	0.997–1.002
Panel 3: Air pollutants on severe obesity (*d* = 2, polynomial = 3)^c^							
OR(SO)	1.069***	1.035***	1.013***	0.994***	0.999***	1.005	1.004
95%CI	1.014–1.208	1.015–1.055	1.007–1.019	0.991–0.997	0.998–0.999	0.998–1.012	0.996–1.007

^a^ Individuals were defined as OW, OB, and SO if their BMI ≥ 24, BMI ≥28, and BMI ≥30, respectively. AQI was air quality index, OW was overweight, OB was obesity, and SO was severe obesity. ^b^ Selected the sample where *d* = 2, polynomial = 3 to report. All the covariates were controlled by command ‘covs’ in the ‘rdrobust,’ using a fuzzy RD model with an instrumental variable of whether the individual was located north of the QHB. ^c^*p < 0.05, **p < 0.01, ***p < 0.001.

The influence of specific air pollutants varied as follows:

PM_2.5_ and PM_10_ positively influenced OW/OB/SO. More specifically, a 1 μg/m^3^ increase in PM2.5 was associated with increases in OW (17.3%, OR = 1.173, 95% CI = 1.094–1.252), OB (2.2%, OR = 1.022, 95% CI = 1.016–1.028), and SO (3.4%, OR = 1.035, 95% CI = 1.015–1.055). Similarly, a 1 μg/m^3^ increase in PM10 led to significant increases in OW (5.3%, OR = 1.053, 95% CI = 1.030–1.076), OB (0.8%, OR = 1.008, 95% CI = 1.006–1.010), and SO (2%, OR = 1.013, 95% CI = 1.007–1.019).

SO2 and CO had a negative impact on the prevalence of OW/OB/SO. A 1 μg/m^3^ increase in SO2 was associated with decreases in OW (2.8%, OR = 0.972, 95% CI = 0.965–0.979), OB (0.3%, OR = 0.997, 95% CI = 0.996–0.998), and SO (0.6%, OR = 0.994, 95% CI = 0.991–0.997). Additionally, a 1 μg/m^3^ increase in CO was linked to reductions in OW (1.4%, OR = 0.986, 95% CI = 0.980–0.992), OB (0.2%, OR = 0.998, 95% CI = 0.997–0.999), and SO (0.1%, OR = 0.999, 95% CI = 0.998–0.999), all statistically significant.

#### 3.2.3 Heterogeneity analysis

Table 3 present the heterogeneous relationship between APCs and obesity outcomes across different subgroups. The five valid APCs (AQI, PM_2.5_, PM_10_, SO_2_, and CO) had a more pronounced impact on men than women, on older individuals (Age ≥ 40) compared to young individuals (Age ≤ 40), and in rural areas compared to urban areas.

**Table 3:** Heterogeneous causal direct relationship (odds ratio and 95% CI) between air pollution (AQIs) and BMI^a^ across gender^b^, age^c^ and registration^d^: RD estimates.^e^

Variables	AQI	PM_2.5_	PM_10_	SO_2_	CO	NO_2_	O_3_
Panel 1: Air pollutants on overweight (*d* = 2, polynomial = 3)^f^							
Male	1.245***	1.261***	1.062***	0.947***	0.971***	1.051	1.031
Female	1.103***	1.089**	1.049*	0.987	0.995	1.039	1.014
Youth	1.008***	1.064***	1.015*	0.999	0.999	1.006	1.002
Aged	1.126***	1.073***	1.131***	0.969***	0.975***	1.050	1.040
Rural	1.104***	1.146***	1.065***	0.997***	0.999***	1.003	1.000
Urban	1.219***	1.261***	1.062*	0.947	0.971	1.051	1.031
Panel 2: Air pollutants on obesity (*d* = 2, polynomial = 3)^f^							
Male	1.198***	1.351***	1.043***	0.996***	1.012***	1.038	1.008
Female	0.912	1.011*	1.003*	1.009	0.984	1.006	1.002
Youth	1.073***	1.043**	1.016	1.011	1.014	1.011	1.000
Aged	1.095***	1.051**	1.046**	0.963***	0.966***	1.075	1.021
Rural	1.212***	1.017**	1.039***	0.975***	0.969***	0.999	0.990
Urban	0.932	1.010	1.015	0.999	0.999	1.027	1.009
Panel 3: Air pollutants on severe obesity (*d* = 2, polynomial = 3)^f^							
Male	1.217***	1.115***	1.014***	0.997**	0.985**	1.012	1.004
Female	0.996	1.009	1.008	0.999	0.999	0.998	1.001
Youth	0.994	1.010	1.010	0.999	0.999	1.002	1.003
Aged	1.073***	1.022**	1.028**	0.986**	0.977**	1.011	1.007
Rural	0.995	1.021**	1.024**	0.998*	0.975**	1.028	1.013
Urban	1.126***	1.013*	1.009	0.999	0.999	0.995	1.001

^a^Individuals were defined as OW, OB, and SO if their BMI ≥ 24, BMI ≥28, and BMI ≥30, respectively. AQI was air quality index, OW was overweight, OB was obesity, and SO was severe obesity. ^b^Where gender = 1 is male and gender = 0 is female. ^c^Where age = 1 is aged group and age = 0 is young group. ^d^Where registration = 1 is rural group and registration = 0 is urban group. ^e^Selected the sample where d = 2, polynomial = 3 to report. All the covariates were controlled by command ‘covs’ in the ‘rdrobust,’ using a fuzzy RD model with an instrumental variable of whether the individual was located north of the QHB. ^f^*p < 0.05, **p < 0.01, ***p < 0.001.

### 3.3 The potential mechanism between air pollutants and obesity outcomes: SEM model

**Figure 2:**
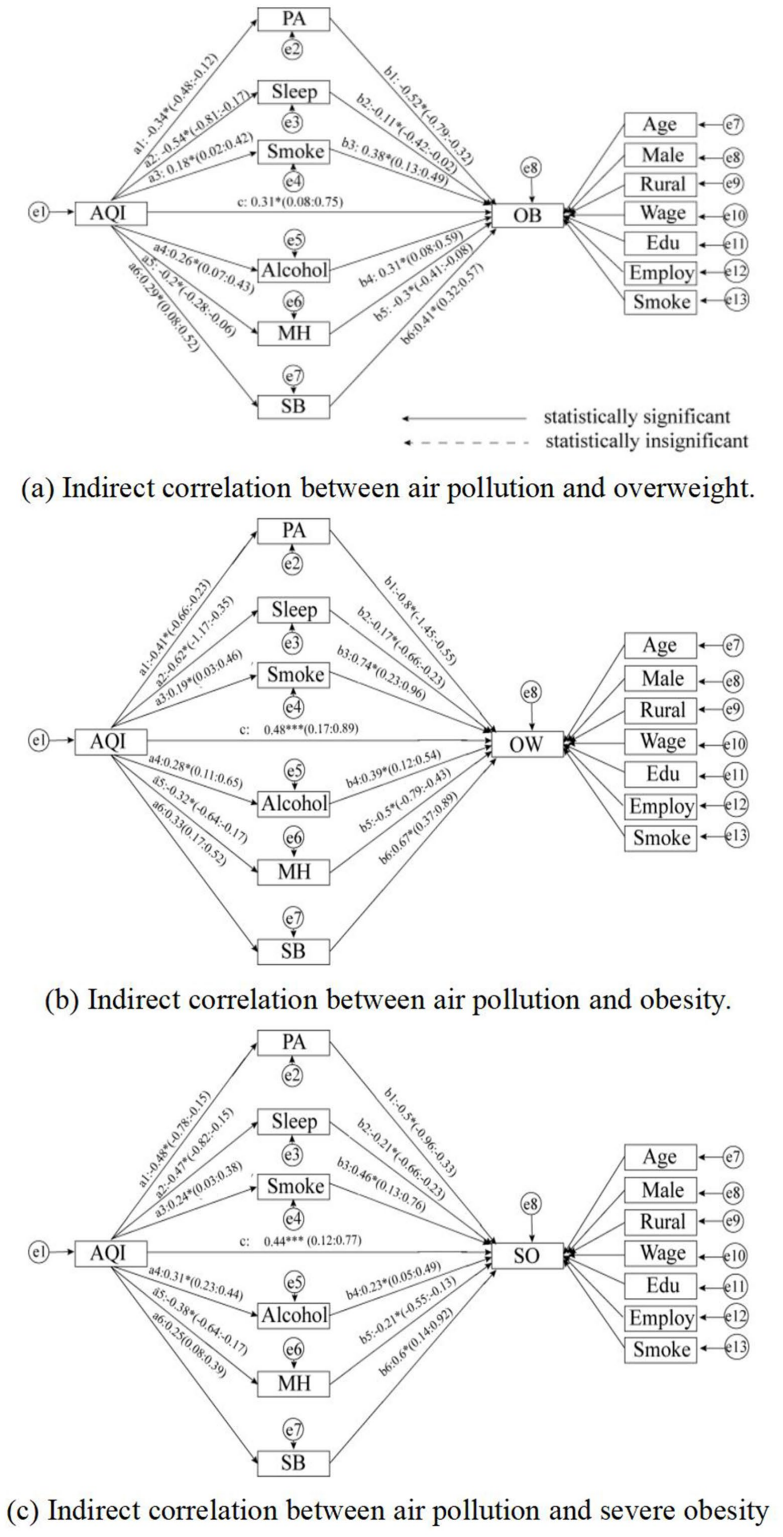
The indirect correlation between air pollution and BMI. The endogeneity problem generated by statistical bias was balanced by RD. The endogeneity problem generated by statistical bias was balanced by RD, and the SEM model was constructed using MPlus 8.9. RMSEA = 0.037, GFI = 0.962, CFI = 0.864, ACFI = 0.951 and PGFI = 0.736. [* = *p* < 0.05, ** = *p* < 0.01, *** = *p* < 0.001, (−) = *p* > 0.05].

Using the SEM approach, we employed multiple parallel mediators, including PA, sleep, smoking, alcohol consumption, MH, and SB, to explore the potential mechanisms between air pollution and OW/OB/SO from a social behavior perspective. For a clearer explanation, we focused solely on AQI; the results are shown in Table 4 and Figure 2a-c. The following results were captured:

1. Social behavior factors significantly mediated the relationship between air pollution and OB, accounting for 64.71% of the mediating effect in OW (*β* = 0.88, 95%CI = 0.27–1.15), 65.17% in OB (*β* = 0.58, 95%CI = 0.14–1.09), and 62.17% in SO (*β* = 0.74, 95%CI = 0.27–1.36). These proportions were notably higher than the direct effects, indicating that social behavior factors are the primary lens through which the obesogenic environment should be understood.
2. All six specific social behavior factors (PA, sleep, smoking, alcohol consumption, MH, and SB) significantly mediated the effects of air pollution on OB. Among these, PA and SB had relatively higher mediating influences, while sleep, smoking, alcohol, and MH had relatively lower mediating effects.

**Table 4:** The indirect correlation between air pollution (AQI)^a^ and obesity: the SEM mediation from the social behavior perspective.^b^

Influences	Independent variable = OW^c^	Independent variable = OB^c^	Independent variable = SO^c^
Panel 1: Full model			
(a1) AQI → PA	−0.41*(−0.66:-0.23)	−0.34***(−0.48:-0.12)	−0.48*(−0.78:-0.15)
(b1) PA → OB	−0.8*(−1.45:-0.55)	−0.52***(−0.79:-0.32)	−0.5*(−0.96:-0.33)
(a2) AQI → Sleep	−0.62*(−1.17:-0.35)	−0.54***(−0.81:-0.17)	−0.47*(−0.82:-0.15)
(b2) Sleep→OB	−0.17*(−0.66:-0.23)	−0.11***(−0.42:-0.02)	−0.21*(−0.66:-0.23)
(a3) AQI → Smoke	0.19*(0.03:0.46)	0.18***(0.02:0.42)	0.24*(0.03:0.38)
(b3) Smoke→OB	0.74*(0.23:0.96)	0.38***(0.13:0.49)	0.46*(0.13:0.76)
(a4) AQI → Alcohol	0.28*(0.11:0.65)	0.26***(0.07:0.43)	0.31*(0.23:0.44)
(b4) Alcohol→OB	0.39*(0.12:0.54)	0.31***(0.08:0.59)	0.23*(0.05:0.49)
(a5) AQI → MH	−0.32*(−0.64:-0.17)	−0.2***(−0.28:-0.06)	−0.38*(−0.64:-0.17)
(b5) MH → OB	−0.5*(−0.79:-0.43)	−0.3***(−0.41:-0.08)	−0.21*(−0.55:-0.13)
(a6) AQI → SB	0.33 (0.17:0.52)	0.29***(0.08:0.52)	0.25(0.08:0.39)
(b6) SB → OB	0.67*(0.37:0.89)	0.41***(0.32:0.57)	0.6*(0.14:0.92)
(c) AQI → OB	0.48***(0.17:0.89)	0.31***(0.08:0.75)	0.44*** (0.12:0.77)
Panel 2: SEM model of influences from AQI to OB			
Total^d^	1.36***(0.39:2.14)	0.89***(0.31:1.65)	1.18***(0.49:1.73)
Dir.Total^e^	0.48***(0.17:0.89)	0.31***(0.08:0.75)	0.44*** (0.12:0.77)
Ind.Total^f^	0.88[64.71%]*** (0.27:1.15)	0.58[65.17%]^g^*** (0.08:0.75)	0.74[62.17%]*** (0.27:1.36)
Ind1 (a1 × b1)	0.33[24.26%]***^g^ (0.02:0.59)	0.18[31.03%)^g^* (0.04:0.29)	0.24***[32.43%]^g^ (0.08:0.45)
Ind2 (a2 × b2)	0.11[8.09%]***^g^ (0.07:0.14)	0.06[10.34%)^g^* (0.02:0.13)	0.1***[13.51%]^g^ (0.07:0.12)
Ind3 (a3 × b3)	0.14[10.29%]*** (0.03:0.28)^g^	0.07[12.06%)^g^* (0.02:0.15)	0.11***[14.86%]^g^ (0.05:0.23)
Ind4 (a4 × b4)	0.11[8.09%]*** (0.04:0.15)^g^	0.08[13.79%)^g^* (0.04:0.11)	0.07***[9.46%]^g^ (0.02:0.11)
Ind5 (a5 × b5)	0.16[11.76%]*** (0.03:0.42)^g^	0.06[10.34%)^g^* (0.01:0.09)	0.08***[10.81%]^g^ (0.02:0.13)
Ind6 (a6 × b6)	0.22[16.18%]*** (0.11:0.59)^g^	0.12[20.69%)^g^* (0.04:0.16)	0.15***[20.27%]^g^ (0.06:0.25)
Panel 3: SEM model fitting			
Fit index^h^	RMSEA = 0.06, CFI = 0.85, TLI = 0.89	RMSEA = 0.04, CFI = 0.93, TLI = 0.91	RMSEA = 0.07, CFI = 0.81, TLI = 0.84

^a^We focused only on AQI to reveal the potential relationship between air pollution and obesity in general. AQI was air quality index, OW was overweight, OB was obesity, SO was severe obesity, PA was physical activity, MH was mental health, and SB was sedentary behavior. ^b^“()” was the 95% CI, “[]” was the proportion of mediating influences. ^c^OW and SO were BMI ≥24 and BMI ≥30, respectively. ^d^*p < 0.05, **p < 0.01, ***p < 0.001. ^e^Total influences refers to the sum of all influences. ^f^Direct influences were the influences between air pollution and obesity after excluding the influence of mediations. ^g^Indirect influences were air pollution and obesity through mediating factors. ^h^Model fitting values including RMSEA where <0.05 is acceptable and the smaller, the better, CFI where >0.08 is acceptable and the bigger, the better, TLI where >0.09 is acceptable and the bigger, the better.

### 3.4 Robustness

**Figure 3:**
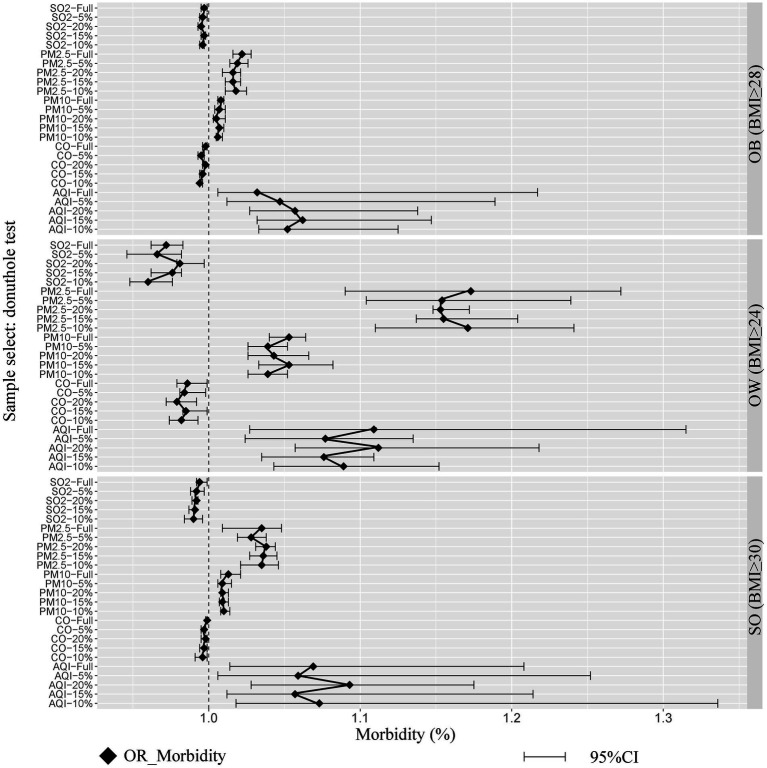
Robustness tests: the donut hole test. Whose principle was ‘the closer the sample to the accordingly, we removed 5, 10, 15, and 20% of the samples near the breakpoints sequentially.’ The endogeneity problem generated by statistical bias was balanced by RD, and the SEM model was constructed using MPlus 8.9. RMSEA = 0.037, GFI =0.962, CFI =0.864, ACFI =0.951 and PGFI = 0.736.

We primarily examined the robustness of the direct correlation, given that the SEM fit was satisfactory. To test robustness, we employed a special testing tool in RD analysis known as the donut-hole test, which operates on the principle that “the closer the sample to the breakpoints, the more likely it is to be manipulated.” We sequentially removed 5, 10, 15, and 20% of the samples closest to the breakpoints, and the results are presented in Figure 3. The estimated causal relationship remained significant after removing 5, 10, 15, and 20% of the samples around the QHB. Therefore, the findings of direct correlation in the present study were robust. Additional information on the donut-hole test is provided in Supplementary Table S5.

## 4 Discussion

This large-scale cohort study, based on nationally representative data, provides a comprehensive analysis of the direct and indirect relationships between air pollution and OW/OB/SO. Our findings address an existing academic gap and contribute to a deeper understanding of the obesogenic environment. The following conclusions were drawn:

1. Air pollution is generally positively associated with BMI. The AQI index was found to have a positive relationship with BMI, with a 1-unit increase in AQI correlating with a 10.9, 3.2, and 6.9% increase in the prevalence of OW, OB, and SO, respectively. We provided causal evidence for the effects of air pollution on obesity using a more rigorous statistical method, which is consistent with several cross-country analyses (1, 2, 6). Our study, however, estimated a stronger influence, which may be attributed to two factors: (1) our estimation was based on a Chinese sample, where air pollution and obesity are more severe, and (2) our results were based on causal estimation, which isolated the net relationship between air pollution and individual BMI.
2. PM_2.5_ and PM_10_ positively affected BMI, while SO2 and CO had a negative effect. In contrast, the results suggested a positive influence of a 1 μg/m^3^ increase in PM_2.5_ concentration on the morbidity of OW, OB, and SO by 17.3, 2.2, and 3.5%, respectively, and positive influences of a 1 μg/m^3^ increase in PM_10_ concentration related to the rise of morbidity of OW, OB, and SO by 5.3, 0.8, and 1.3%, respectively. PM is composed of various chemicals, with polycyclic aromatic hydrocarbons (PAHs) being the most harmful component for obesity (5, 27, 29).
3. PAHs were clinically proven to increase *β*-2 microglobulin, leading to impaired glomerular filtration, renal dysfunction, and metabolic system disorder (8). On the other hand, the RD model revealed that a 1 μg/m^3^ increase in SO_2_ concentration was significantly associated with a 2, 0.3, and 0.6% decrease in OW, OB, and SO morbidity, respectively, and a 1 μg/m^3^ increase in CO concentration was significantly associated with a 1.4, 0.2, and 0.1% decrease in the OW, OB, and SO morbidity, respectively. Several prior clinical studies provided the pathological insight that SO_2_ was composed of substantial sulfur elements, a major component of amino acids, which may have contributed to the maintenance of oxygen supply balance, promoted metabolism, and increased daily calorie consumption (34). Meanwhile, moderate amounts of CO could have regulated vascular tension, eliminated vascular inflammation, and reduced fat accumulation in the blood (7). Notably, previous research on the correlation between SO_2_, CO, and obesity was limited; only two out of a representative meta-analysis of 18 studies investigated the influence of SO_2_ and CO on OW/OB (35).
4. The influence of air pollution on obesity outcomes was more significant in male, older, and rural groups compared to their female, younger, and urban counterparts. First, the results of the subgroup analysis revealed that males were more likely to gain weight due to air pollution compared to females. Females are systematically less exposed to outdoor work due to physical disadvantages, which, in turn, create disparities in air pollution exposure between males and females, further exacerbating gender inequality in BMI (36). Second, aged individuals were more susceptible to gaining weight from air pollution compared to younger individuals. Exposures to air pollution induced a greater inflammatory response in the adipose tissue of aged individuals, as their respiratory system deteriorated, and were more likely to bind to polycyclic aromatic hydrocarbons (PAHs) in air pollution, which led to severe metabolic degradation and, ultimately, systemic adipose tissue growth and gain weight (37). Third, the rural group was more likely to gain weight due to air pollution compared to the urban group. Rural China tended to lag behind in urbanization and economic development with lower air pollution, but the impact of air pollution on obesity was higher among rural than urban residents. This had been previously assessed and validated by researchers using satellite-based Random Forest Models (RFMs) for causal assessment (38). The scenario suggested that rural residents in China were not resilient to the adverse health outcomes of air pollution. Considering that rural populations already live in a more hazardous obesogenic environment, specialized economic, psychosocial, and medical interventions are needed to enhance their resilience to the risk of BMI.
5. The potential mechanism between air pollution and obesity was systematically mediated by social behavior determinants. The six social behavior factors collectively mediated more than 60% of the association between air pollution and BMI consistently across the OW/OB/SO samples. This finding suggests that social behavior is the predominant determinant in shaping adverse health outcomes. Cockerham’s seminal health lifestyle theory is useful in explaining the influence of social behavior on obesity, as it posits that collective patterns of social behavior are shaped by the norms, values, and material resources consistent with an individual’s living situation or life chances, connecting them with others in similar social strata (25).

After adjusting the width and polynomial order in the RD estimation, the direct association between air pollution and OW/OB remained robust, even when subjected to the donut sensitivity test. The good fit of the SEM model further supports the robustness of the potential mechanisms linking air pollution to OW/OB identified in this study.

Based on the results of the present study, we propose the following policy recommendations:

1. Building a joint prevention and control policy framework that addresses both air pollution and BMI: The present study identified air pollution as a significant obesogenic factor, highlighting the necessity of jointly preventing obesity and air pollution. We urge all stakeholders to collaboratively develop a comprehensive policy framework to mitigate the risks associated with both obesity and air pollution. This framework should include the use of air quality monitors, the implementation of scientifically based environmental prevention methods, and the enforcement of air quality standards in accordance with WHO guidelines and national policies (2, 11, 21, 40).
2. Focusing on BMI disparities influenced by social determinants: We recommend that governments develop policy frameworks that integrate biological susceptibility and socioeconomic factors to effectively address overweight and obesity. Particular attention should be given to protecting vulnerable populations, such as low-income, rural, and older adult individuals (18, 38, 39).

The current study presents a comprehensive analysis of the relationship between air pollution and BMI; however, several limitations should be carefully considered:

1. Statistical ecological bias: This investigation captures only city-level relationships between air pollution and obesity morbidity, limiting the generalization of the findings to the individual level.
2. APC measurement issues: Potential bias may arise from the use of electric heating in certain cities north of the QHB, such as Beijing and Tianjin, which may reduce variation in air pollution exposure and thus underestimate the impact of air pollution on obesity outcomes. Although efforts were made to address this bias by employing an optimal bandwidth, it remains a concern.
3. Neglect of short-term exposure identification: The current study exclusively examines the long-term influences of air pollution exposure on obesity outcomes and does not account for short-term exposures. This is particularly relevant since short-term exposures may cause significant metabolic system disorders and induce obesity, especially among vulnerable populations such as infants, pregnant women, and patients.
4. Measurement bias: The survey data relied on self-reported information regarding physical characteristics, which may lead to underestimation or overestimation of height or weight. Additionally, this self-reported information may introduce recall bias, affecting the accuracy of BMI calculations.

Given the conclusions and limitations of this study, we propose the following directions for future research:

1. Identifying causal associations between obesogenic environments and individual BMI using more accurate cohort data: Future studies should employ large-scale analyses that account for individual-level factors and use causal statistical methods such as propensity score matching or instrumental variables to mitigate potential biases.
2. More accurate estimation of obesogenic environments through randomized controlled trials (RCTs): Future research should consider using RCTs to simulate quasi-randomized distributions of obesogenic environments, such as air pollutants.
3. Considering both long-term and short-term air pollution exposure through an integrated analytical framework: To comprehensively understand the relationship between air pollution and obesity, future investigations should examine evidence from both short-term and long-term exposures while also comparing the disparities between them.

## 5 Conclusion

This study provides a comprehensive analysis of the relationship between air pollution and obesity outcomes using nationally representative data from China. The findings indicate that air pollution is generally positively associated with BMI, with PM_2.5_ and PM_10_ having positive effects and SO_2_ and CO showing negative effects. Furthermore, the impact is more significant in men, older adults, and rural populations compared to their female, younger, and urban counterparts. Additionally, the potential correlation between air pollution and obesity is systematically mediated by social behavior determinants. These results have several significant implications for understanding the obesogenic environment, particularly in highlighting the need to integrate obesity prevention strategies with air pollution control efforts.

## Data Availability

The datasets presented in this study can be found in online repositories. The names of the repository/repositories and accession number(s) can be found at: https://www.isss.pku.edu.cn/cfps/.

## Footnotes

^1^www.tianditu.gov.cn

## Supplementary material

SUPPLEMENTARY FIGURE S1 The initial SEM model of the potential mechanism between air pollution and overweight. (The endogeneity problem generated by statistical bias was balanced by RD, and the SEM model was constructed using MPlus 8.2.)

SUPPLEMENTARY FIGURE S2 RD plot: polynomial = 1. (The result was shown through the ‘RD the plot’ command in STATA 16.0; the significance of differences was tested through the *t*-test, and P4 denoted polynomial = 1.)

SUPPLEMENTARY FIGURE S3 RD plot: polynomial = 2. (The result was shown through the ‘RD plot’ command in STATA 16.0; the significance of differences was tested through the *t*-test, P4 denoted polynomial = 2.)

SUPPLEMENTARY FIGURE S4 RD plot: polynomial = 3. (The result was shown through the ‘RD plot’ command in STATA 16.0; the significance of differences was tested through the *t*-test, and P4 denoted polynomial = 3.)
