# Supplementary figures and images for: How air pollution influences the difference between overweight and obesity: a comprehensive analysis of direct and indirect correlations
Source: Front Public Health. 2024 Nov 1;12:1403197. doi: 10.3389/fpubh.2024.1403197 (PMC11566261; doi:10.3389/fpubh.2024.1403197)

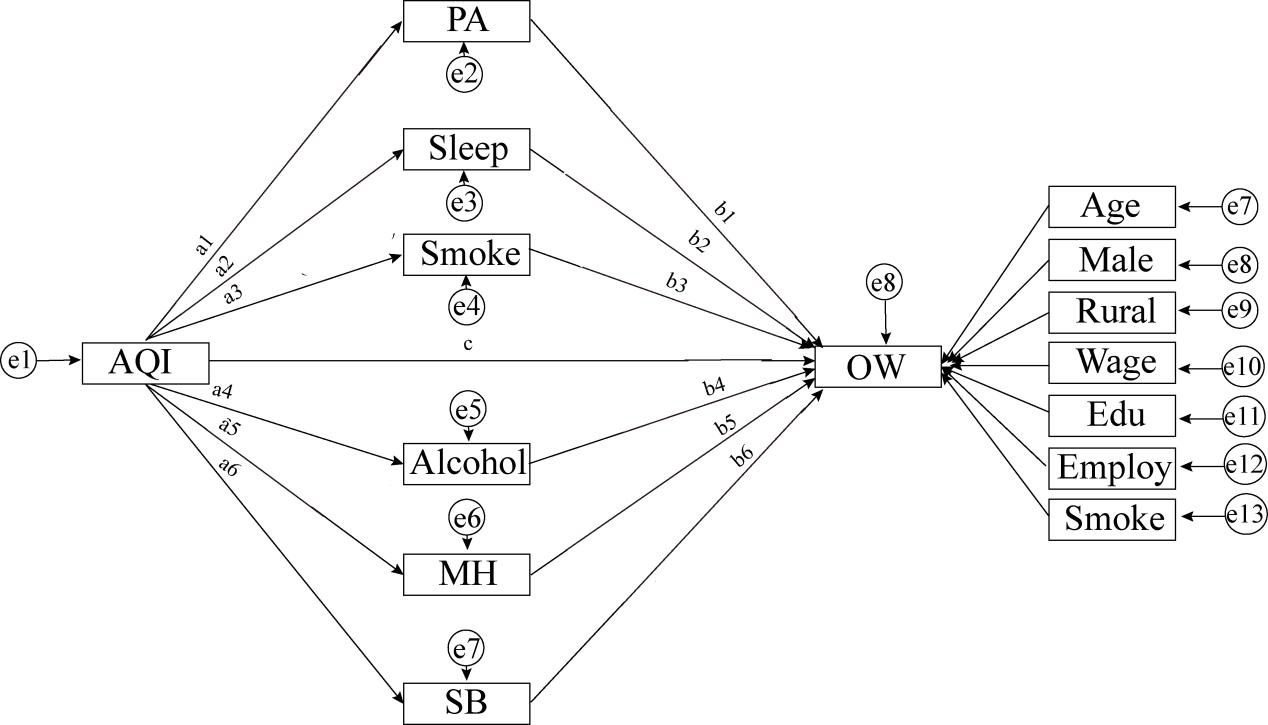

Supplement: SUPPLEMENTARY FIGURE S1 — The initial SEM model of the potential mechanism between air pollution and overweight. (The endogeneity problem generated by statistical bias was balanced by RD, and the SEM model was constructed using MPlus 8.2.) [file Image_1.jpeg]

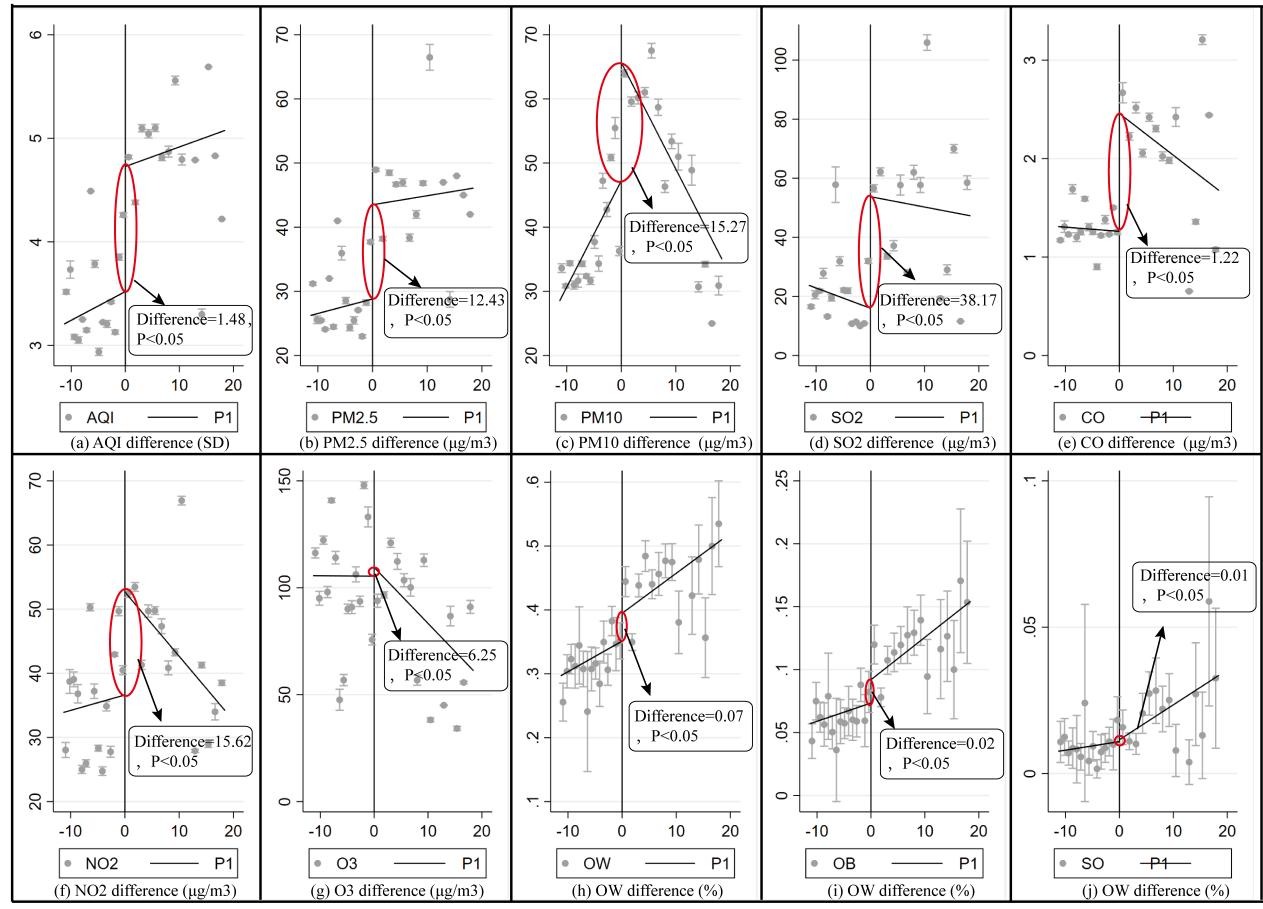

Supplement: SUPPLEMENTARY FIGURE S2 — RD plot: polynomial = 1. (The result was shown through the ‘RD the plot’ command in STATA 16.0; the significance of differences was tested through the t-test, and P4 denoted polynomial = 1.) [file Image_2.jpeg]

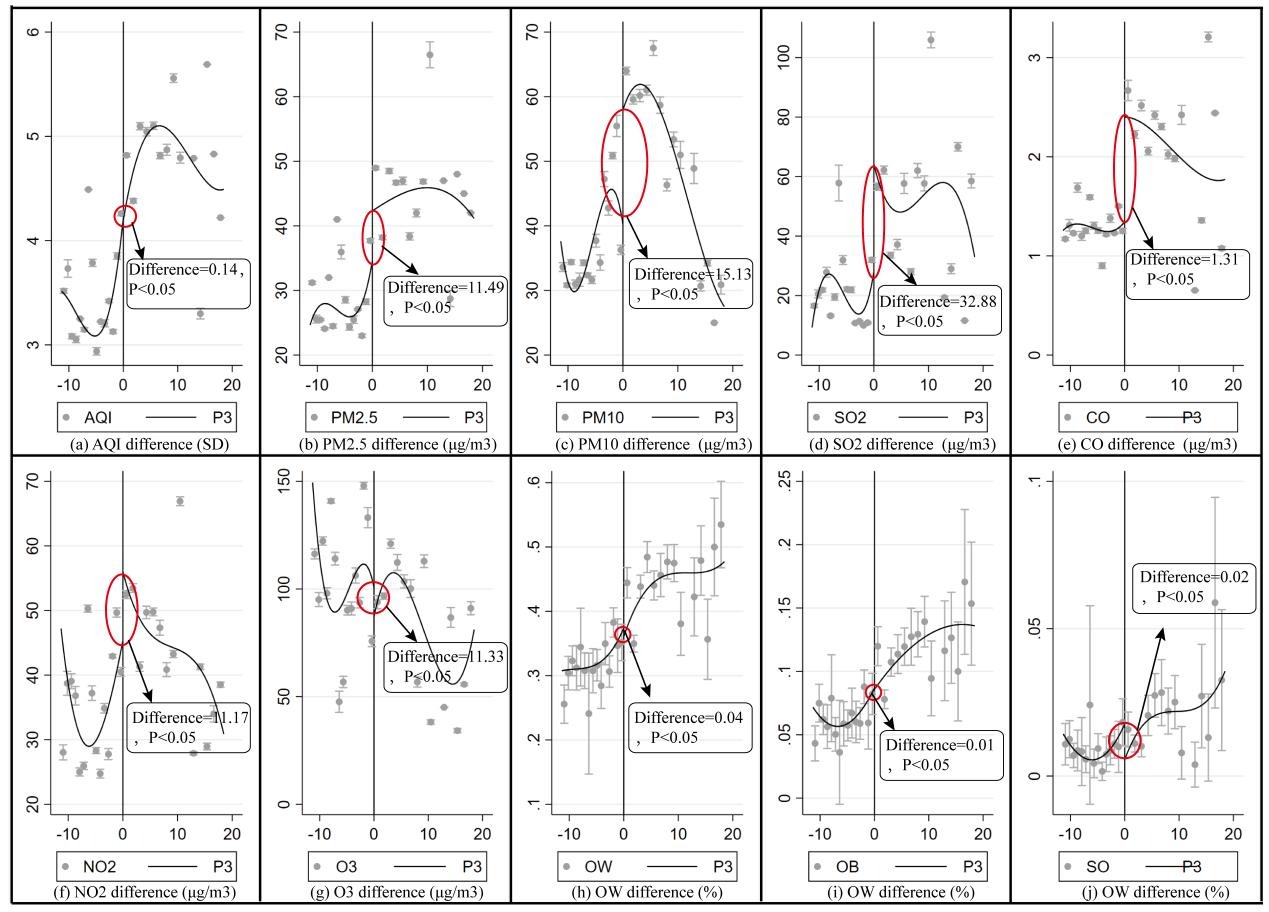

Supplement: SUPPLEMENTARY FIGURE S3 — RD plot: polynomial = 2. (The result was shown through the ‘RD plot’ command in STATA 16.0; the significance of differences was tested through the t-test, P4 denoted polynomial = 2.) [file Image_3.jpeg]

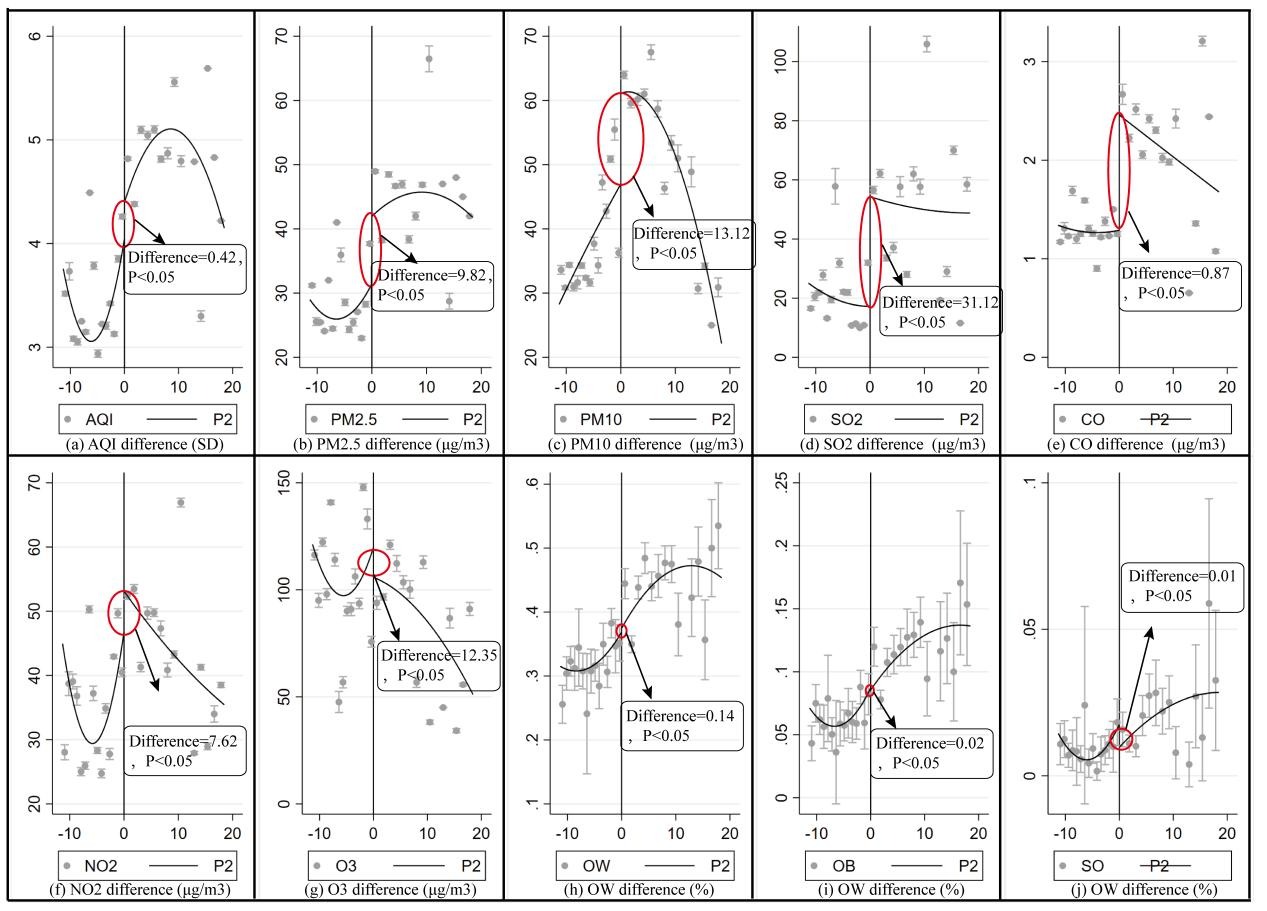

Supplement: SUPPLEMENTARY FIGURE S4 — RD plot: polynomial = 3. (The result was shown through the ‘RD plot’ command in STATA 16.0; the significance of differences was tested through the t-test, and P4 denoted polynomial = 3.) [file Image_4.jpeg]
