# Supplementary material for: How air pollution influences the difference between overweight and obesity: a comprehensive analysis of direct and indirect correlations
Source: Front Public Health. 2024 Nov 1;12:1403197. doi: 10.3389/fpubh.2024.1403197 (PMC11566261; doi:10.3389/fpubh.2024.1403197)
Supplement: Supplementary file 5 [file Table_1.docx]

Table ST1 Each residence and its QHB distance (100KM) information.

| CityID | QHB-D | CityID | QHB-D | CityID | QHB-D |
| --- | --- | --- | --- | --- | --- |
| Zhanjiang | -11.305 | Xuancheng | -2.443 | Zibo | 3.646 |
| Maoming | -10.867 | hangzhou | -2.379 | Wuwei | 3.716 |
| Yangjiang | -10.780 | Ganzi | -2.307 | Handan | 3.722 |
| Fangchenggang | -10.573 | Wuhan | -2.252 | Xingtai | 4.192 |
| Zhuhai | -10.482 | Chengdu | -2.107 | texas | 4.434 |
| Jiangmen | -10.128 | Shanghai | -1.872 | Yantai | 4.562 |
| Simao | -10.102 | Deyang | -1.567 | Hengshui | 4.753 |
| Yunfu | -9.928 | Hefei | -1.215 | Taiyuan | 5.150 |
| Dongguan | -9.725 | Wuxi | -1.195 | Shijiazhuang | 5.166 |
| Guangzhou | -9.598 | Xinyang | -0.694 | Cangzhou | 5.243 |
| Shantou | -9.402 | Longnan | -0.422 | Luliang | 5.350 |
| Jieyang | -9.322 | Guangyuan | -0.275 | Xinzhou | 5.660 |
| Wuzhou | -9.092 | Yangzhou | -0.159 | Yulin | 5.793 |
| Qingyuan | -8.995 | Zhumadian | 0.151 | Dalian | 5.956 |
| Meizhou | -8.647 | Nanyang | 0.295 | Tianjin | 6.042 |
| Yuxi | -8.463 | Xiangfan | 0.623 | Langfang | 6.462 |
| Shaoguan | -7.955 | Luohe | 0.746 | Beijing | 6.899 |
| Guilin | -7.224 | Zhoukou | 0.755 | Qinhuangdao | 6.949 |
| Ganzhou | -7.027 | Pingdingshan | 0.978 | Datong | 7.304 |
| Putian | -7.018 | Xuchang | 1.220 | Huludao | 7.754 |
| red river | -7.005 | Linxia | 1.440 | Yingkou | 7.789 |
| Dali | -7.005 | Shangqiu | 1.490 | Zhangjiakou | 7.874 |
| Yongzhou | -6.402 | Lianyungang | 1.672 | Danton | 7.959 |
| Ningde | -5.805 | Zaozhuang | 1.761 | Jinzhou | 8.116 |
| Hengyang | -5.791 | Xi'an | 1.841 | Anshan | 8.465 |
| Gian | -5.778 | Tianshui | 1.860 | Liaoyang | 8.582 |
| Fuzhou | -5.478 | Kaifeng | 1.930 | Chaoyang | 8.588 |
| Qiandongnan | -5.193 | Zhengzhou | 1.958 | benxi | 8.784 |
| Qiannan | -5.093 | Luoyang | 1.966 | Fuxin | 9.042 |
| Qianxinan | -4.993 | Dingxi | 2.026 | Shenyang | 9.153 |
| Loudi | -4.905 | Weinan | 2.033 | Tieling | 9.764 |
| Xiangtan | -4.847 | Lanzhou | 2.033 | Tonghua | 10.087 |
| Zunyi | -4.693 | Qingyang | 2.123 | Siping | 10.758 |
| Changsha | -4.528 | Yuncheng | 2.424 | Matsubara | 12.801 |
| Liangshan | -4.122 | Jiaozuo | 2.483 | Harbin | 13.995 |
| Yibin | -3.889 | Xinxiang | 2.491 | Daqing | 14.216 |
| Yueyang | -3.368 | sunshine | 2.525 | Jixi | 15.909 |
| Leshan | -3.235 | silver | 2.622 | Hegang | 17.171 |
| Ningbo | -3.198 | Pingliang | 2.842 | Heihe | 18.471 |
| Meishan | -3.007 | Laiwu | 3.082 | chongqing | -2.965 |
| Anqing | -2.543 | Anyang | 3.239 | Changzhi | 3.422 |

^a^ QHB-D<0 means the residence is located in the south of QH, QHB>0 means the north.

^b^ QHB-D Determined by the vertical distance from the point (Residence) to the line (QHB) in ARCGIS.
