# Supplementary material for: How air pollution influences the difference between overweight and obesity: a comprehensive analysis of direct and indirect correlations
Source: Front Public Health. 2024 Nov 1;12:1403197. doi: 10.3389/fpubh.2024.1403197 (PMC11566261; doi:10.3389/fpubh.2024.1403197)
Supplement: Supplementary file 6 [file Table_2.docx]

Tabel ST2. The AQI standards and formula ranges in China.^a^

| AQI limit values^e^ | Air pollution limit values^bc^ | | | | | |
| --- | --- | --- | --- | --- | --- | --- |
|  | PM_2.5_ | PM_10_ | SO_2_ | CO | NO_2_ | O_3_ |
| 0 | 0 | 0 | 0 | 0 | 0 | 0 |
| 50 | 35 | 50 | 50 | 2 | 40 | 100 |
| 100 | 75 | 150 | 150 | 4 | 80 | 160 |
| 150 | 115 | 250 | 475 | 14 | 180 | 215 |
| 200 | 150 | 350 | 800 | 24 | 280 | 265 |
| 300 | 250 | 420 | 1600 | 36 | 565 | 800 |
| 400 | 350 | 500 | 2100 | 48 | 750 | 1000 |
| 500 | 500 | 600 | 2620 | 60 | 940 | 1200 |

^a^ Suppose the PM_2.5_ concentration in an area of China is 55 µg/m³. According to the standard, the corresponding concentration range is 35-75, and the AQI index range is 50-100. According to the equation S1, the AQI = [(100-50)/(75-35)]*(55-35)+50=75.

^b^ PM_2.5_, PM_10_, SO_2_, NO_2_, CO calculated by 24-hour average concentration.

^c^ O_3_ based on 8-hour average concentration.
