# Supplementary material for: How air pollution influences the difference between overweight and obesity: a comprehensive analysis of direct and indirect correlations
Source: Front Public Health. 2024 Nov 1;12:1403197. doi: 10.3389/fpubh.2024.1403197 (PMC11566261; doi:10.3389/fpubh.2024.1403197)
Supplement: Supplementary file 7 [file Table_3.docx]

Table ST3. Manipulation of the Obesity outcomes, APCs information and other socio-demographic characteristics of the Chinese adults in CFPS.

| Variables | Operationalization | Source |
| --- | --- | --- |
| OW | Respondents with BMI ≥ 24 | CFPS^a^ |
| OB | Respondents with BMI ≥ 28 | CFPS^a^ |
| SB | Respondents with BMI ≥ 32 | CFPS^a^ |
| AQI | Air quality in the respondent's location, higher denoted more polluted | CESY^b^ and CCAQMR^c^ |
| PM_2.5_ | Annual average PM_2.5_ concentration | CESY^b^ and CCAQMR^c^ |
| PM_10_ | Annual average PM_10_ concentration | CESY^b^ and CCAQMR^c^ |
| SO_2_ | Annual average SO_2_ concentration | CESY^b^ and CCAQMR |
| CO | Annual average CO concentration | CESY^b^ and CCAQMR^c^ |
| NO_2_ | Annual average NO_2_ concentration | CESY^b^ and CCAQMR^c^ |
| O_3_ | Annual average O_3_ concentration | CESY^b^ and CCAQMR^c^ |
| Age | Age of Respondents | CFPS^a^ |
| Gender | Gender of Respondents, 0 is female and1 is male | CFPS^a^ |
| Registration | Registration, 0 is urban and 1 is rural | CFPS^a^ |
| Wage | Total wage for the last whole year | CFPS^a^ |
| Edu | Total years of education completed by the respondent | CFPS^a^ |
| Employed | Employment Status of Respondents, 0 is unemployed, 1 is employed | CFPS^a^ |
| Smoke | Does the respondent smoke, 0 is non-smoke and 1 is smoke | CFPS^a^ |
| PA | Respondents' frequency of exercise per week. | CFPS^a^ |
| Sleep | Respondents' average daily sleep time | CFPS^a^ |
| MH | Respondents' mental health, calculated by CES-D 9 scale.^d^ | CFPS^a^ |
| Alcohol | Whether the respondent drinks alcohol, 0=non-alcohol, 1=alcohol | CFPS^a^ |
| SB | Respondents' average daily sedentary time. | CFPS^a^ |
| Tem | Average annual temperature | CESY^b^ |
| Wind | Annual average wind | CESY^b^ |

^a^ CFPS avaliable at www.isss.pku.edu.cn/cfps/.

^b^ CESY avaliable at www.stats.gov.cn/ztjc/ztsj/hjtjzl/.

^c^ CCAQMR avaliable at https://www.mee.gov.cn/hjzl/dqhj/cskqzlzkyb/.

^d^ Center for Epidemiological Studies Depression (CES-D 9) was a commonly used scale to measure mental health.
