# Supplementary material for: How air pollution influences the difference between overweight and obesity: a comprehensive analysis of direct and indirect correlations
Source: Front Public Health. 2024 Nov 1;12:1403197. doi: 10.3389/fpubh.2024.1403197 (PMC11566261; doi:10.3389/fpubh.2024.1403197)
Supplement: Supplementary file 8 [file Table_4.docx]

Table ST4. Robustness test: compare the AIC information^a^ of the selected model^b^ with other models^c^.

| Model selected | OW | OB | SO | Total^d^ |
| --- | --- | --- | --- | --- |
| RD, d=2 | 5249 | 1895 | 645 | 7789 |
| RD, d=5 | 5877 | 1958 | 683 | 8518 |
| RD, d=8 | 6233 | 2103 | 711 | 9047 |
| RD, d=10 | 5781 | 1955 | 729 | 8465 |

^a^ AIC is a measure of Goodness of fit, the smaller the value the better the model.

^b^ RD estimates where d = 2 and polynomial order = 4.

^c^ Other RD estimates where d = 5 and polynomial order = 4, d = 8 and polynomial order = 4, d = 10 and polynomial order = 4

^d^ Total AIC value is the sum of OW, OB and SO.
