# Supplementary material for: How air pollution influences the difference between overweight and obesity: a comprehensive analysis of direct and indirect correlations
Source: Front Public Health. 2024 Nov 1;12:1403197. doi: 10.3389/fpubh.2024.1403197 (PMC11566261; doi:10.3389/fpubh.2024.1403197)
Supplement: Supplementary file 9 [file Table_5.docx]

Tabel ST5. Identifying whether the samples near the breakpoint (QHB) were sensitive: the donuthole test (odds ratio and 95% CI).^a,c^

| Sample | OR | 95%CL | 95%CU | OR | 95%CL | 95%CU | OR | 95%CL | 95%CU |
| --- | --- | --- | --- | --- | --- | --- | --- | --- | --- |
|  | OW (BMI≥24)^b^ | | | OB (BMI≥28)^b^ | | | SO (BMI≥30)^b^ | | |
| AQI-Full | 1.109 | 1.027 | 1.315 | 1.032 | 1.006 | 1.217 | 1.069 | 1.014 | 1.208 |
| AQI-5% | 1.077 | 1.024 | 1.135 | 1.047 | 1.012 | 1.189 | 1.059 | 1.006 | 1.252 |
| AQI-10% | 1.089 | 1.043 | 1.152 | 1.052 | 1.033 | 1.125 | 1.073 | 1.018 | 1.336 |
| AQI-15% | 1.076 | 1.035 | 1.109 | 1.062 | 1.032 | 1.147 | 1.057 | 1.012 | 1.214 |
| AQI-20% | 1.112 | 1.057 | 1.218 | 1.057 | 1.027 | 1.138 | 1.093 | 1.028 | 1.175 |
| PM_2.5_-Full | 1.173 | 1.090 | 1.272 | 1.022 | 1.016 | 1.028 | 1.035 | 1.009 | 1.048 |
| PM_2.5_-5% | 1.154 | 1.104 | 1.239 | 1.019 | 1.014 | 1.026 | 1.028 | 1.019 | 1.038 |
| PM_2.5_-10% | 1.171 | 1.110 | 1.241 | 1.018 | 1.011 | 1.025 | 1.035 | 1.021 | 1.046 |
| PM_2.5_-15% | 1.155 | 1.137 | 1.204 | 1.016 | 1.011 | 1.021 | 1.036 | 1.027 | 1.045 |
| PM_2.5_-20% | 1.153 | 1.148 | 1.172 | 1.016 | 1.009 | 1.021 | 1.038 | 1.031 | 1.044 |
| PM_10_-Full | 1.053 | 1.040 | 1.064 | 1.008 | 1.006 | 1.010 | 1.013 | 1.008 | 1.021 |
| PM_10_-5% | 1.039 | 1.026 | 1.052 | 1.007 | 1.004 | 1.011 | 1.009 | 1.006 | 1.015 |
| PM_10_-10% | 1.039 | 1.026 | 1.052 | 1.006 | 1.005 | 1.009 | 1.010 | 1.008 | 1.014 |
| PM_10_-15% | 1.053 | 1.033 | 1.082 | 1.007 | 1.006 | 1.010 | 1.009 | 1.007 | 1.013 |
| PM_10_-20% | 1.043 | 1.026 | 1.066 | 1.005 | 1.003 | 1.011 | 1.009 | 1.008 | 1.013 |
| SO_2_-Full | 0.972 | 0.983 | 0.962 | 0.997 | 0.995 | 0.999 | 0.994 | 0.992 | 0.999 |
| SO_2_-5% | 0.966 | 0.946 | 0.982 | 0.996 | 0.994 | 0.999 | 0.992 | 0.988 | 0.997 |
| SO_2_-10% | 0.960 | 0.948 | 0.976 | 0.996 | 0.994 | 0.997 | 0.990 | 0.984 | 0.996 |
| SO_2_-15% | 0.976 | 0.962 | 0.982 | 0.997 | 0.995 | 0.998 | 0.991 | 0.987 | 0.991 |
| SO_2_-20% | 0.981 | 0.979 | 0.997 | 0.995 | 0.993 | 0.996 | 0.992 | 0.989 | 0.993 |
| CO-Full | 0.986 | 0.979 | 0.999 | 0.998 | 0.996 | 0.999 | 0.999 | 0.998 | 0.999 |
| CO-5% | 0.984 | 0.981 | 0.998 | 0.995 | 0.993 | 0.997 | 0.997 | 0.995 | 0.998 |
| CO-10% | 0.982 | 0.974 | 0.993 | 0.994 | 0.994 | 0.996 | 0.996 | 0.991 | 0.999 |
| CO-15% | 0.985 | 0.983 | 0.999 | 0.996 | 0.994 | 0.995 | 0.997 | 0.994 | 0.999 |
| CO-20% | 0.979 | 0.972 | 0.992 | 0.998 | 0.996 | 0.997 | 0.998 | 0.995 | 0.999 |

^a^ The donuthole test to further test the robustness, whose principle was ”the closer the sample near to the breakpoint, the more likely it is to be manipulated”. Accordingly, we removed 5%, 10%, 15%, and 20% of the samples near the breakpoints, sequentially.

^b^ Individuals were defiende as OW, OB, SO if their BMI ≥ 24, BMI ≥28, BMI ≥30, respectively.

^c^ The selected RD model was d = 2 and polynomial order = 4.
